# c-IAP1 Binds and Processes PCSK9 Protein: Linking the c-IAP1 in a TNF-α Pathway to PCSK9-Mediated LDLR Degradation Pathway

**DOI:** 10.3390/molecules171012086

**Published:** 2012-10-15

**Authors:** Weiming Xu, Lizhi Liu, David Hornby

**Affiliations:** The Krebs Institute, Department of Molecular Biology and Biotechnology, University of Sheffield, Firth Court, Western Bank, Sheffield S10 2TN, UK; Email: Lizhi.liu@sheffield.ac.uk (L.L.); d.hornby@shef.ac.uk (D.H.)

**Keywords:** c-IAP1, PCSK9, TNF-α, LDLR, Cholesterol, K27, ubiquitination, E3 ligase, Stub1

## Abstract

Recent genetic studies have shown that PCSK9, one of the key genes in cholesterol metabolism, plays a critical role by controlling the level of low-density lipoprotein receptor. However, how PCSK9 mediates LDLR degradation is still unknown. By combining a shotgun proteomic method and differential analysis of natural occurring mutations of the PCSK9 gene, we found that an E3 ubiquitin ligase c-IAP1 binds and processes PCSK9 protein**.** One of the ‘gain-of-function’ mutations, S127R, is defective with respect to binding to c-IAP1, and thus has defective autocatalytic activity. Knockdown of c-IAP1 impairs PCSK9 processing and autocatalytic cleavage. In *c-IAP1 null* mouse embryonic fibroblasts (*MEFs*), there is a dramatic decrease in secreted mature PCSK9 protein accompanied by a significant increase in LDLR protein levels compared with matched wild-type MEF cells. c-IAP1 also acts as an E3 ligase for ubiquitination of PCSK9. Ubiquitin containing only lysine-27 mediated PCSK9 ubiquitination by c-IAP1. Given K27-linked polyubiquitination promotes lysosomal localization, the finding indicates the c-IAP1 acts on both secretion of PCSK9 and its lysosomal localization. The novel pathway described here will open new avenues for exploring novel disease treatments.

## 1. Introduction

Proprotein convertase subtilisin/kexin type 9 (PCSK9) is encoded by one of three genes implicated in autosomal dominant hypercholesterolaemia (ADH), in addition to LDLR (low-density lipoprotein receptor) and APOB (apolipoprotein B). Recent genetic studies have shown that PCSK9 plays a critical role in cholesterol metabolism, by controlling the level of low-density lipoprotein receptor [1,2]. A wide spectrum of mutations in the PCSK9 gene has been found in the human population. PCSK9 gain-of function variations are associated with hypercholesterolemia, whereas loss-of-function variations are associated with hypocholesterolemia [3,4].

PCSK9 belongs to a family of secretory serine proteinases known as proprotein convertases (PCs) [5]. It is synthesized as a 73 kDa zymogen and undergoes autocatalytic cleavage in the endoplasmic reticulum and it is secreted as a 63 kDa mature protein, which forms a complex with the N-terminal predomain [5]. To date, most studies of PCSK9 suggest it acts as a chaperone for LDLR and its related receptors, by directing them to the lysosomes for degradation. However, the precise mechanism by which PCSK9 escorts LDLR to lysosomes is still unknown. It has been suggested there are other interacting proteins, or co-receptors, which can associate with PCSK9 and LDLR to escort them into intracellular targets, such as endosomes, lysosomes or the cell surface [6,7].

In order to identify these proposed PCSK9 binding proteins, we have generated a FLAG-tagged PCSK9 expressing cell line and have used anti-FLAG antibody affinity purification and shotgun proteomic analysis to identify potential binding partners of PCSK9 and to study their roles in PCSK9-mediated LDLR degradation.

## 2. Results and Discussion

### 2.1. Identification of Potential PCSK9 Binding Proteins through Affinity Purification and Shotgun LC-MS/MS Analysis

To identify novel binding partners of PCSK9, we generated a human T-Rex-293 stable cell line overexpressing FLAG-tagged wild-type PCSK9 [8] and PCSK9 carrying ADH mutations (p.S127R, D374Y, F216 L). The FLAG-tagged wild-type PCSK9 and associated proteins were isolated by performing anti-FLAG immunoprecipitation (IP) from cellular extracts; the eluted protein mixture was then subjected to shotgun proteomic analysis [9]. Briefly, protein complexes pulled down by immunoprecipitation with FLAG*-*tagged PCSK9 protein, were subjected to limited electrophoresis, after which 3–5 molecular weight regions were cut out and digested. Liquid chromatography/mass spectrometry/mass spectrometry (LC-MS/MS) was performed on each fraction. The lists of identified proteins for each sample (with their scores) were subjected to statistical validation and aligned for comparison using the Scaffold program. A negative control cell line, T-Rex-293 cells transfected with empty vector, pcDNA3.1, was used for background subtraction.

In total, 22 co-IP proteins were identified (Table 1) that contain at least two unique spectra (two distinct peptides) in three independent experimental samples but were absent (zero spectra) in all three negative control samples. Many of these proteins are ER-localised proteins, such as the UDP-glucose glycoprotein glucosyltransferase 1 (UGGT-1), protein disulfide isomerase family A member 4 (PDIA4, also called endoplasmic reticulum resident protein 72, ERP72) and calmegin (CLGN). Some of the identified proteins are associated with the ubiquitination pathway, such as c-IAP1, TRAF2 and Stub1 E3 ligase. Others are molecular chaperones, such as DNJA1, DNJA2, DNJA3, DJB11, and DJC10 in the DnaJ (Hsp40) homolog subfamily. We also identified several mitochondrial carriers, including SLC25 A1, A10 and A12.

**Table 1:** List of the potential PCSK9 binding proteins identified through affinity purification and shotgun LC-MS/MS analysis. Each protein was identified by at least two matched spectra (95% confidence minimum) in all three experiments (exp) with no spectrum identified in the control samples (empty vector, emp).

Gene Symbols	Gene Names	Experiment 1		Experiment 2		Experiment 3	
		emp	exp	emp	exp	emp	exp
PCSK9	proprotein convertase subtilisin/kexin type 9	3	1466	2	1171	4	1128
UGGT1	UDP-glucose glycoprotein glucosyltransferase 1	0	9	0	12	0	15
PDIA4 (ERP72)	protein disulfide isomerase family A, member 4	0	7	0	5	0	7
SLC25A1	solute carrier family 25 member 1	0	6	0	2	0	6
DNAJA1	DnaJ (Hsp40) homolog, subfamily A, member 1	0	12	0	10	0	7
CLGN	Calmegin	0	2	0	2	0	2
DNAJA2	DnaJ (Hsp40) homolog, subfamily A, member 2	0	2	0	3	0	4
c-IAP1 (BIRC2)	cellular inhibitor of apoptosis protein 1	0	7	0	9	0	8
TBB6	tubulin, beta 6	0	5	0	2	0	3
TRAF2	TNF receptor-associated factor 2	0	4	0	3	0	3
SLC25A10	solute carrier family 25. member 10	0	6	0	3	0	6
DNAJB11	DnaJ (Hsp40) homolog, subfamily B, member 11	0	5	0	4	0	3
CDIPT	CDP-diacylglycerol-inositol 3-phosphatidyltransferase	0	3	0	6	0	4
DNAJC10	DnaJ (Hsp40) homolog, subfamily C, member 10	0	6	0	3	0	6
DNAJA3	DnaJ (Hsp40) homolog, subfamily A, member 3	0	2	0	3	0	3
RHOT1	ras homolog gene family, member T1	0	2	0	3	0	4
AGK	Acylglycerol kinase lipid kinase	0	2	0	3	0	2
HSPB1	heat shock 27kDa protein 1	0	4	0	5	0	2
RCN1	reticulocalbin 1	0	3	0	4	0	5
Ribosomal L28	partial ribosomal protein L28 variant	0	2	0	6	0	5
Stub1	STIP1 homology and U-box containing protein 1	0	2	0	3	0	2
PGRC1	progesterone receptor membrane component 1	0	2	0	2	0	2
SLC25A12	Solute carrier family 25 member 12	0	2	0	5	0	4

### 2.2. c-IAP1/TRAF2 Complex Binds PCSK9

**Figure 1:**
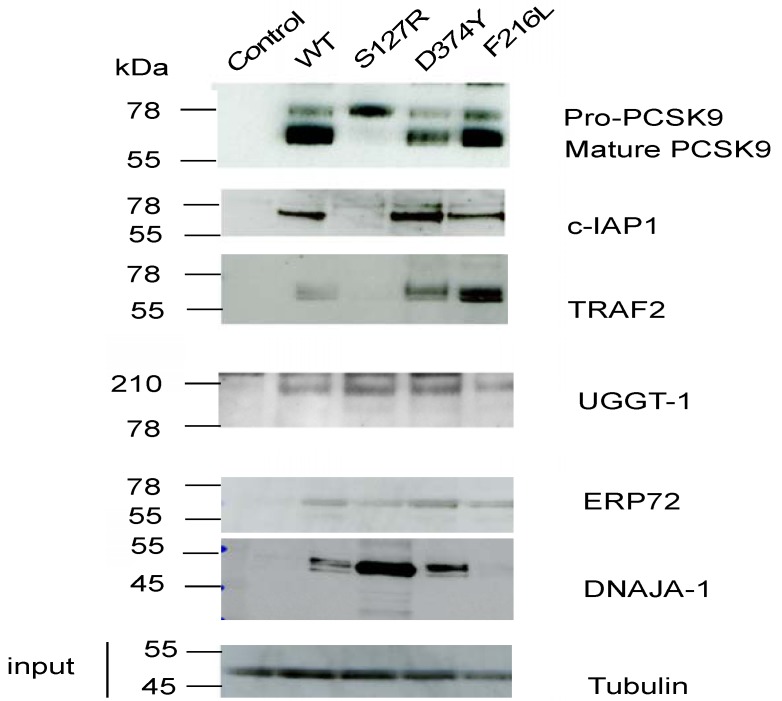
Immunoprecipitation (IP)/western blot analysis of novel PCSK9 binding proteins in the PCSK9-FLAG pull-down assay. Equal amount of cellular extracts from a T-Rex 293 cell line stably overexpressing FLAG-tagged wild-type PCSK9 or PCSK9 carrying ADH mutations (p.S127R, D374Y, F216 L) and a negative control cell line, T-Rex-293 cells transfected with the empty vector pcDNA3.1(control) were subjected to anti-FLAG IP and Western blots probed with the antibodies indicated. 5% of the cell lysates prior to IP were used for the input, probed with anti-α-tubulin antibody. The western blots shown are representative of three separate experiments.

We further confirmed five of the 22 PCSK9 binding proteins by Western blotting using commercial antibodies (Figure 1). Among the five proteins confirmed by Western blot (c-IAP1, TRAF2, UGGT-1, ERP72 and DNAJA-1), we observed that one of the “gain-of-function” mutants, PCSK9-S127R, which has impaired autocatalytic activity, was defective in binding to the c-IAP1 and TRAF2 proteins (Figure 1). c-IAP1 and TRAF2 are known binding partners in the TNF-α-mediated apoptosis pathway [10].

**Figure 2:**
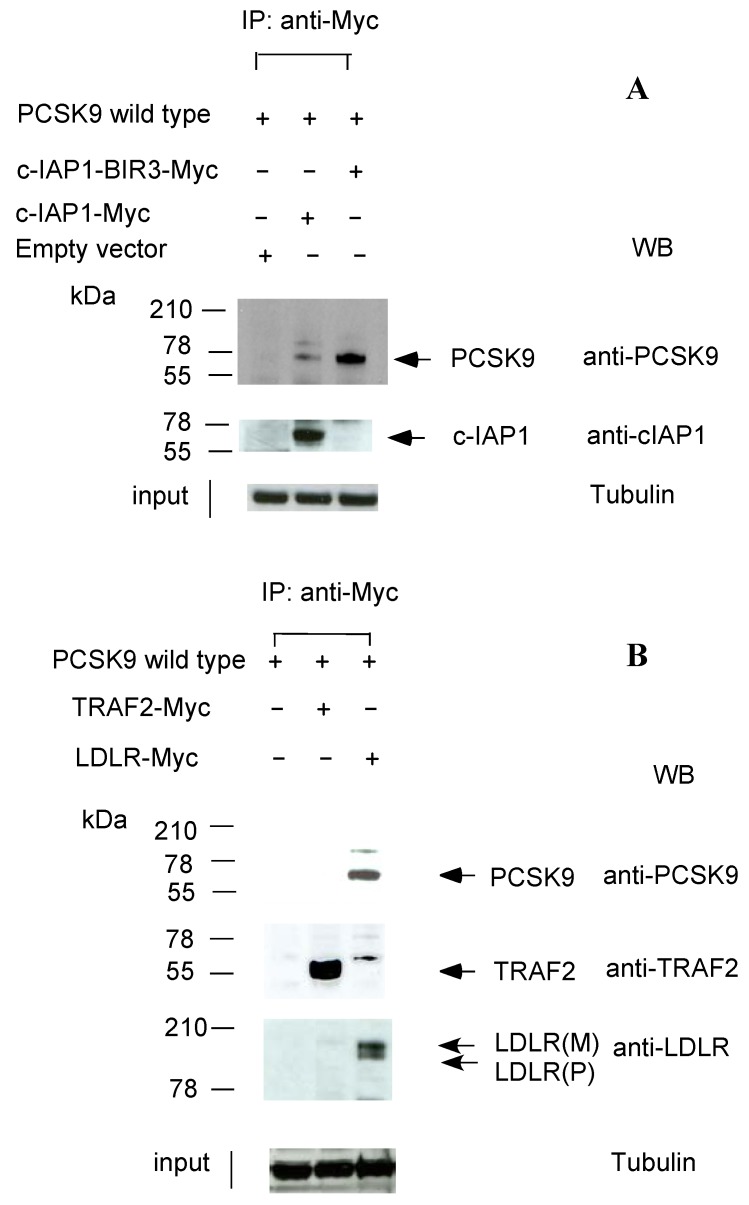
c-IAP1 interacts with PCSK9. (**A**) Co-IP of PCSK9-wt and c-IAP1. Myc-tagged c-IAP1 or c-IAP1-BIR3 was co-transfected with either empty vector (pCMV6-entry vector) or FLAG-tagged wild-type PCSK9 into T-Rex-293 cells. Cell lysates were immunoprecipitated with an anti-myc tag antibody followed by immunoblotting with either anti-PCSK9 or anti-c-IAP1 antibodies; (**B**) Co-IP of PCSK9-wt, TRAF2 and LDLR. Myc-tagged TRAF2 or LDLR was co-transfected with FLAG-tagged wild-type PCSK9 into T-Rex-293 cells. Cell lysates were immunoprecipitated with an anti-myc tag antibody followed by immunoblotting with either anti-PCSK9 or anti-TRAF or LDLR antibodies. The Western blots shown are representative of three separate experiments.

To further confirm the binding of c-IAP1 to wild-type PCSK9, we co-transfected wild-type FLAG-tagged PCSK9 (pCMV-PCSK9-FLAG) and myc-tagged cIAP-1 cloned into a pCMV6 expression vector (Origene Inc., Rockville, MD, USA) into T-Rex-293 cells. The empty vector, pCMV6-entry vector (Origene, Inc.), was used as a control vector. After 24 h, the cell lysates were immunoprecipitated with anti-myc antibody. As shown in Figure 2A, the PCSK9 protein was detected in the IP pellet (Lane 2). To determine which regions of c-IAP1 were involved in binding to PCSK9, we made several deletion mutant constructs of c-IAP1 for PCSK9 binding studies. We found that only the region containing the baculoviral IAP repeat 3 (BIR3) domain of c-IAP1 could be used to pull down wild-type PCSK9 (Figure 2A, lane 3), indicating that c-IAP1-BIR3 is the binding site for PCSK9. The binding of PCSK9 to c-IAP1 was further confirmed using surface plasmon resonance (SPR) experiments, in which the binding affinities of the wild-type PCSK9 protein (isolated from the whole cell extract) for c-IAP1 were determined at pH 7.4 with a Kd of 44.3 ± 5 nM (n = 3, T100 evaluation software, Supplementary Figure S1). We also used confocal microscopy to conduct immunostaining studies to confirm the colocalisation of PCSK9 and c-IAP1 in the cytoplasm of 293 cells with stable overexpression of PCSK9 (Supplementary Figure S2).

We next determined whether TRAF2 binds to wild-type PCSK9. We co-transfected wild-type FLAG-tagged PCSK9 and myc-tagged TRAF2-pCMV6 expression vectors into T-Rex-293 cells. After 24 h, the cell lysates were immunoprecipitated with anti-myc antibody. We were unable to detect the PCSK9 protein in the IP pellet (Figure 2B, lane 2), indicating that TRAF2 was not a direct binding partner for PCSK9. c-IAP1 was thus the only physical binding partner of PCSK9 in the c-IAP1/TRAF2 complex. As a positive control, we co-transfected wild-type FLAG-tagged PCSK9 and myc-tagged LDLR-pCMV6 into T-Rex-293 cells. After 24 h, the cell lysates were immunoprecipitated with an anti-myc antibody. We were able to detect the PCSK9 protein in the IP pellet (Figure 2B, lane 3), confirming LDLR binding to PCSK9 in our system.

### 2.3. c-IAP1 Knock-Down

**Figure 3:**
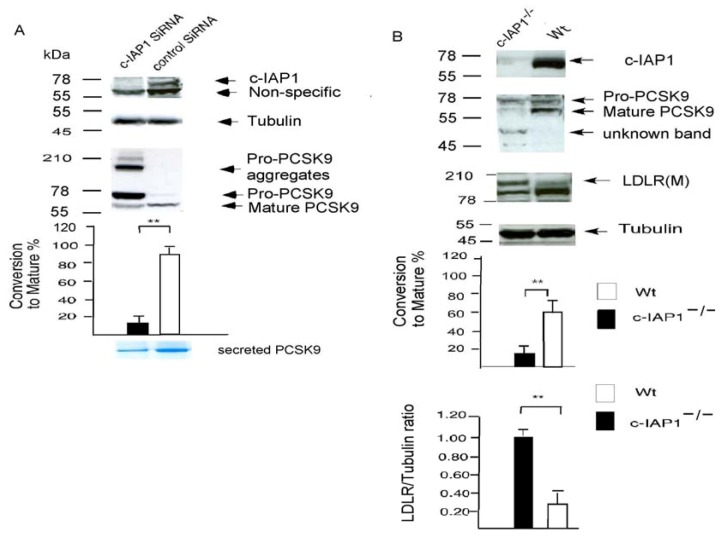
c-IAP1 Knock-down. (**A**) A wild-type PCSK9 overexpressed T-Rex-293 stable cell line was transfected with pGB c-IAP1 siRNA and a Western blot was probed with anti-c-IAP1 or anti-PCSK9 antibodies. Scanning densitometry analysis of three western blots is shown below. Data are presented as the percentage conversion to mature PCSK9 (p63), calculated as the p63 value divided by the sum of p63 + p75 (Pro-PCSK9), divided by the tubulin, multiplied by 100. ** indicates a significant difference (*p* = 0.009) from c-IAP siRNA treated cells from control siRNA cells. Lower panel: Coomassie-Blue-stainedsecreted PCSK9 isolated from the 100mL of cultured medium over 2 days from c-IAP1 siRNA treated or control siRNA treated samples; (**B**) Western blot analysis of the LDLR and PCSK9 protein in c-IAP1-deficient (c-IAP1^−/−^, reference 11) MEFs and the matched wild-type (Wt) MEFs. The percentage conversion to mature PCSK9 level was calculated as described above. LDLR amounts were quantified and normalised to the amount of α-tubulin in three experiments. The ratio of LDLR/tubulin in c-IAP1 deficient cells was assigned a value of 1.00. ** Indicates a significant difference (*p* < 0.01) between c-IAP1 deficient MEF cells and matched wild-type MEF cells.

We next used a siRNA-c-IAP1 construct to knock down endogenous c-IAP 1 in a human T-Rex-293 stable cell line that overexpressed FLAG-tagged wild-type PCSK9. We observed significantly increased pro-PCSK9 species (90% PCSK9 protein is still pro-PCSK9, only 10% is converted to the mature species) in comparison to the non-silencing RNA control (over 90% of PCSK9 is converted to the mature species), indicating that c-IAP1 is directly involved in processing PCSK9 from the proprotein to the functionally mature protein (Figure 3A). We detected much less secreted mature PCSK9 protein in medium collected over 2 days from c-IAP1 siRNA treated *versus* control siRNA treated samples (in 100 mL medium, siRNA treated cells (1 × 10^7^ cells) yielded 20 µg protein, whereas the control siRNA treated sample yielded 100 µg protein (1 × 10^7^ cells) (Figure 3A, lower panel). We have also detected high-molecular weight aggregates of the pro-PCSK9 protein in c-IAP1 siRNA cells (Figure 3A).

We then used c-IAP1 null mouse embryonic fibroblasts (MEFs [11]) to analyse PCSK9 processing. As shown in Figure 3B, only 17% pro-PCSK9 is converted to the mature polypeptide in c-IAP1 knockout MEF cells in comparison to the matched wild-type MEF cells, where over 67% of PCSK9 is converted to the mature species. We were unable to detected any secreted PCSK9 in culture supernatants in c-IAP1 null MEFs (1 × 10^7^ cells, ref. [11]) collected on the first day (n = 3) and on the seventh day (n = 3); while in matched wild-type MEFs cells, there was a steady increase in secreted PCSK9 protein in the supernatant (1 × 10^7^ cells) from 1,687 ± 96 pg/mL on the first day (n = 3) and 4,320 ± 450 pg/mL on the seventh day, n = 3). There is also a significant increase in the LDLR protein level (70% ± 15, Figure 3B) in c-IAP1 knockout MEF cells in comparison to matched wild-type MEF cells.

### 2.4. Ubiquitination of PCSK9 by c-IAP1

**Figure 4:**
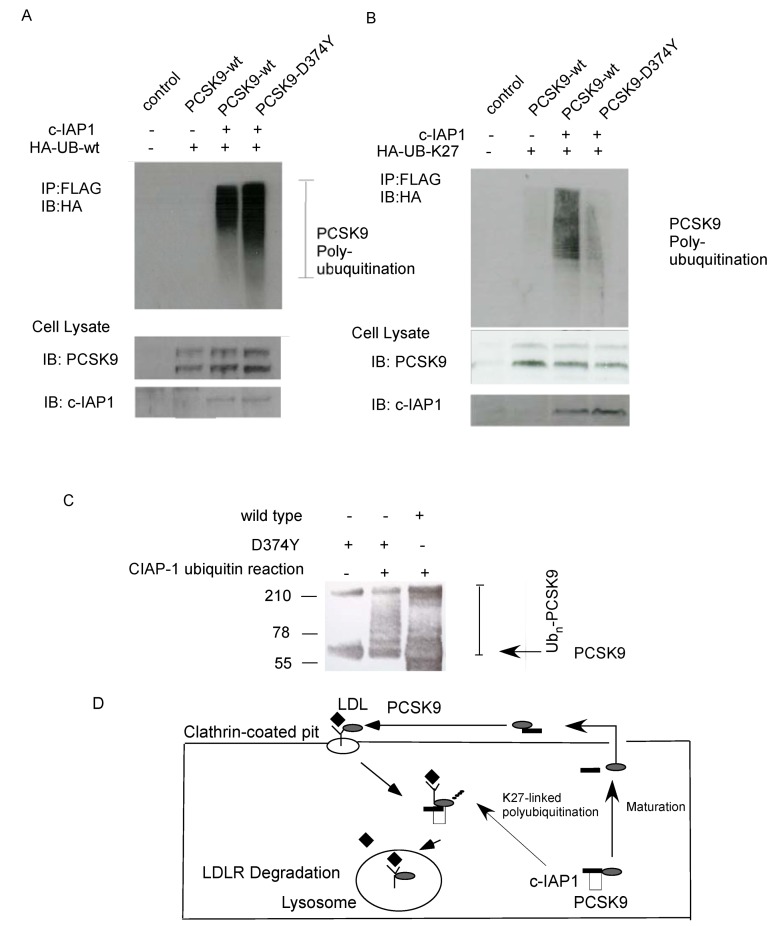
Identification of PCSK9 as a substrate of c-IAP1 ubiquitin ligase *in vivo* and *in vitro*. (**A**) pcDNA.3-Empty vector or HA-tagged wild-type ubiquitin plasmid were co-transfected with FlAG-PCSK9wt or FLAG-PCSK9-D374Y expression vector with or without the pCMV–c-IAP1 plasmids(Image clone 3908352)into T-REX 293 cells. 24h later, the transfected cells were treated with MG132 (10 μM) for 1h, the lysates were immunoprecipitated using the FLAG-immunoprecipitation kit, the elution samples were then analyzed by Western blotting with anti- HA-Tag polyclonal antibody to detect the conjugated HA-ubiquitinated PCSK9.Western blots shown are representative of three separate experiments; (**B**) pcDNA.3-Empty vector or pRK5-HA-Ubiquitin-K27 plasmid were co-transfected with FlAG-PCSK9wt or FLAG-PCSK9-D374Y expression vector with or without the pCMV–c-IAP1 plasmids into T-REX 293 cells. The immunoprecipitation and the western blots analysis were carried out as in A: again, Western blots shown are representative of three separate experiments; (**C**) FLAG-tagged wild-type-PCSK9 and PCSK9-D374Y were subjected to ubiquitination assays in the presence of recombinant c-IAP1. The ubiquitinated PCSK9 was detected by immunoblotting with anti-PCSK9 antibody; (**D**) Proposed model of c-IAP1 and PCSK9 interaction. c-IAP1 can bind to PCSK9 and promote its maturation. c-IAP1 is also targeting the PCSK9 by K27-linked ubiquitination, which lead to the PCSK9/LDLR complex into lysosome, eventually lead to the complex degradation.

As c-IAP1 is a well-known E3 ubiquitin ligase, we then tested c-IAP1’s ability to ubiquitinate PCSK9 *in vivo* and *in vitro*. T-Rex-293 cells were co-transfected with wild-type FLAG-tagged PCSK9 or a ‘gain-of-function’ mutant construct, PCSK9-D374Y-pcDNA3, and pcDNA3.1-(HA-ubiquitin) with or without a CMV-driven c-IAP1 expression vector. After 24 h, whole cell lysates were immunoprecipitated with anti-Flag antibody and probed with anti-HA antibody. As shown in Figure 4A, both the HA-UB/c-IAP1/PCSK9 wild-type PCSK9 and D374 mutant combination resulted in an appearance of multiple high-molecular weight bands representing conjugated HA-Ubiquitinated PCSK9 [12] (Figure 4A, lanes 3 and 4). We have identified lysine 27 on the ubiquitin, as a participant in the ubiquitination of PCSK9 by the LC/MALDI/MS/MS based approaches [13] (Supplementary Figure S3). We have therefore used cells co-transfected with wild-type FLAG-tagged PCSK9 or a ‘gain-of-function’ mutant construct, PCSK9-D374Y-pcDNA3, and pRK5-HA-Ubiquitin-K27 with or without a CMV-driven c-IAP1 expression vector. After 24 h, whole cell lysates were immunoprecipitated with anti-Flag antibody and probed with anti-HA antibody. As shown in Figure 4B, both the wild-type PCSK9 and D374 mutant combination resulted in an appearance of multiple high-molecular weight bands representing conjugated HA-Ubiquitinated PCSK9 (Figure 4B, lanes 3 and 4). For the *in vitro* ubiquitin assay, FLAG-tagged D374Y-PCSK9 and wild-type-PCSK9 were purified and subjected to ubiquitination in the presence or absence of recombinant c-IAP1 (Figure 4C). PCSK9 was ubiquitinated by c-IAP1, as shown by the appearance of multiple high-molecular weight bands on a SDS-PAGE gel representing polyubiquitinated PCSK9 in the presence of recombinant c-IAP1 in both PCSK9-D374Y mutation and wild-type PCSK9 respectively(Figure 4C, lanes 2 and 3).

### 2.5. Discussion

c-IAP1 has two roles in regulating the activities of PCSK9. In its first role, by binding to PCSK9, c-IAP1 promotes its maturation from the proprotein form to the mature form. This conclusion is supported by compelling evidence. One of the ‘gain-of-function’ mutations, S127R, is defective with respect to binding to c-IAP1, and thus has defective autocatalytic activity. Knockdown of c-IAP1 impairs PCSK9 processing and autocatalytic cleavage. In *c-IAP1 null* mouse embryonic fibroblasts (MEFs), there is a dramatic decrease in secreted mature PCSK9 protein, while in the wild-type MEF cells, proprotein form of the PCSK9 was properly processed and secreted into cell supernatants in a reasonable quantity and was functional in mediating degradation of LDLR protein in MEF cells in published data [14] and our own results (Supplementary Figure S4).

The second role of c-IAP1 is its E3 ligase activity in ubiquitinating PCSK9. Both wild-type PCSK9 and PCSK9-D374Y can be ubiquitinated in the *in vivo* and *in vitro* transfection. We found that wild-type PCSK9 polyubiquitination mainly involved lysine residue 27 of ubiquitin (Figure 4B). K27-linked polyubiquitination was shown to promote lysosomal localization of Jun [15]. It is well established the PCSK9 chaperones the LDLR into lysosome. Our results for the first time, highlight the possibility of c-IAP1 targeting the PCSK9 by K27-linked ubiquitination, which then lead to the PCSK9/LDLR complex into lysosome (Figure 4D).

PCSK9 is a endoplasmic/vesicular protein [5], while c-IAP1 is known to localize in the cytoplasm/nucleus. However, recent research has shown that c-IAP1 is also localized in the endoplasmic reticulum and vesicular compartment [16,17]. Wu *et al*. have found that the c-IAP1 was co-localized with ER transmembrane ubiquitin-conjugating enzyme E2 6 (Ubc 6), which served as a cognate E2 for c-IAP1’s E3 activity [16]. The c-IAP1 has also been co-localized with endoplasmic reticulum marker, calreticulin [16]. Calreticulin is a calcium-binding chaperone that is highly concentrated in the lumen of the endoplasmic reticulum [18]. In the TWEAK-FN14 signaling pathway [17], binding of TWEAK to endogenous FN14 recruits a complex containing both cIAP1 and TRAF2 and the complex is recruited to a lysosomal compartment where it is degraded. Consistent with lysosomal degradation, TWEAK-induced TRAF2 degradation is prevented by several different inhibitors of lysosomal function, such as chloroquine and NH_4_Cl [17]. Interestingly, PCSK9 mediated LDLR degradation also occurred via the lysosomal pathway and can be inhibited by same set of lysosomal acidotropic agents such as NH_4_Cl and chloroquine [19]. Therefore, it is possible that c-IAP1 could escort the PCSK9/LDLR complexes to the lysosome (Figure 4D). More recently, c-IAP1 has been shown to directly binds to and processes procaspase-3 and procapspase-7 [20]. The fact that the endoplasmic reticulum is the main site for caspase-3 activation [21] indicates that c-IAP1 being localized in the endoplasmic reticulum and vesicular compartment plays important parts in its diversity roles in the TNF-mediated signal transduction pathway.

However, the precise mechanism by which the S127R mutant proprotein promotes LDLR degradation and causes hypercholesterolaemia remains elusive. The S127 resides in the prodomain. It is strictly conserved within primate, mouse, and rat PCSK9. From our results, this mutation has altered the recognition of the site for c-IAP1 binding and autocatalytic activity, thereby decreasing PCSK9 maturation and secretion in this mutant. Although the autocatalytic process of PCSK9 is necessary for its maturation and secretion, the catalytic activity is not required for secreted PCSK9 to reduce LDLR as a catalytically inactive PCSK9 or a catalytically inert variant of a gain-of-function mutant showed no loss of activity in degrading LDLR [22]. Structural studies suggest that residue 127 is more than 40 Å from the LDL receptor-binding site [23], and overwhelming biochemical studies have shown that the PCSK9-S127R mutant does not have an increased affinity for the LDLR [23,24], In contrast to the other ‘gain of function, D374Y, which has significant higher affinity (5–30 fold) for the LDLR than the wild-type protein [23,24]. One possibility is that S127R could bind to other E3 ligases to regulate the LDLR/PCSK9 intracellular trafficking. In our preliminary shotgun proteomic analysis of S127R *vs.* wild-type PCSK9, an E3 ligase, Stub1 (STIP1homology and U-box containing protein 1), also known as *CHIP* (C terminus of HSC70-Interacting Protein) [25], was found to bind much more strongly to S127R than to wild-type PCSK9 (Xu *et al.*, unpublished data, Supplementary Figure S5). Stub1 is a ubiquitin ligase/co-chaperone that participates in protein quality control by targeting a broad range of chaperone protein substrates, including Hsp70, Hsc70 and Hsp90. Interestingly, Stub1 also mediates the formation of K27-linked ubiquitin chains on Bag1, which stimulates the ability of Bag1 to bind to the proteasome [26]. It is possible that c-IAP1 and Stub1 E3 ligases are both involved in the PCSK9 processing for lysosomal or proteasomal pathways. In addition, another E3 ligase, Inducible degrader of the LDLR (Idol) has been found to be involved in ubiquitination of the LDL receptor [27]. The precise roles of the E3 ligase families in PCSK9-mediated LDLR pathway remain to be explored. Further clarification of this pathway will open completely new avenues for exploring novel treatments for cardiovascular and infectious diseases.

## 3. Experimental

### 3.1. Cell Culture and Immunofluorescence Staining and Image Analysis

HepG2 cells were obtained from the European Cell Culture Collection (Wiltshire, UK). T-Rex 293 cells were obtained from Invitrogen (Paisley, UK). Cells were grown in DMEM containing 25 mM glucose and 10% fetal calf serum, as described [28]. c-IAP1 null mouse embryonic fibroblasts (MEFs) and matched wild-type MEFs cells were kindly provided by Dr. John Silke and grown in DMEM containing 25 mM glucose and 8% fetal calf serum, as described [11]. Fluorescence immunohistochemistry was carried out as previously described [29].

### 3.2. DNA Constructs, Transfections and Western Blot Assays

C-terminal FLAG-tagged Wild-type PCSK9, S127R-PCSK9 and F216L-PCSK9 were kindly provided by Jay D. Horton (University of Texas Southwestern Medical Center, Dallas, TX, USA) [8]. D374Y mutation was introduced by oligonucleotide-direct mutagenesis with forward primer 5'-CATTGGTGCCTCCAGCTACTGCAGCACCTGC-3' and reverse primer 5'-GCAGGTGCTGCAGTAGCTGGAGGCACCAATG-3' using QuickChange XL Mutagenesis kit (Stratagene, La Jolla, CA, USA). The integrity of the construct was confirmed by DNA sequencing. C-terminal MYC-tagged LDLR, cIAP1, TRAF2 in CMV6-based mammalian expression vector were obtained from Origene, Inc. PCR was used to generate deletion constructs of MYC-tagged c-IAP1, containing only BIR1 or BIR2 or BIR3 domains. The PCR products were cloned in frame to the pCMV6 entry clone (Origene, Rockville, MD, USA) with Sgf1 and Mlu1 restriction sites. The successful creation of all constructs was confirmed by DNA sequencing. HA-tagged ubiquitin plasmid (plasmid 18712) and pRK5-HA-Ubiquitin-K27 (plasmid 22902) were purchased from Addgene (Cambridge, MA, USA). CMV-drive c-IAP1 expression vector (Image clone 3908352, pCMV-Sport6) was from Geneserve (Cambridge, UK). All the transfection were done on T-Rex293 cells or HepG2 cells using superfect (Qiagen, Crawley, UK)with 1–2 μg DNA. Cell lysis and western blot analysis were carried out as previous described [28]. The antibodies used were a rabbit antibody directed against amino acids 184–196 of human LDLR (Research Diagnostics Inc. Flanders, NJ, USA) and a rabbit antibody against PCSK9 (Cayman, Ann Arbor, MI, USA). Anti-mouse/human mature LDLR antibody is from BioVision (Milpitas, CA, USA). Other antibodies, including c-IAP1, TRAF2, PDIA4, DNJA1 were from Abcam (Cambridge, UK). To detect mouse c-IAP1, a c-IAP1 antibody, mAb (1E1-1-10) (ENZO, Farmindale, NY, USA) was used to detect the difference between knock-out mouse c-IAP1 MEF and wild-type cells. The antibody was also used to confirm the results from the anti-c-IAP1 antibody from Abcam for human c-IAP1 detection and immunohistochemistry. Goat anti-UGGT-1 antibody was from Santa Cruz Biotechnology (Santa Cruz, CA, USA). Anti-Stub1 antibody and anti-MYC tag antibody 9E10 were from Millipore (Billerica, MA, USA). HA-Tag polyclonal antibody was from Clontech (Mountain View, CA, USA). All transfections were done with either T-Rex 293 cells and HepG2 cells using Superfect (Qiagen). The methods of whole cell extraction and Western blots were carried out as described [28].Western blot densitometry was carried out using the Visionworks LS software (UVP, Cambridge, UK). All data were analysed by the statistical program GB-Stat V5.4 (Dynamic Microsystems, Silver Spring, MD, USA, and SigmaStat, Jandel Scientific, San Rafel, CA, USA). Data were presented as means ± SD, and significance of differences among means was estimated by Student’s *t*-test (two-tailed). *p* values of *<*0.05 were considered statistically significant*.*

### 3.3. Generation of Stable Cell Lines, Immunoprecipitation (IP) and Protein Purification

The PCSK9 expression plasmids were co-transfected with pTK-hygromycin (BD, San Jose, CA, USA) into T-Rex 293 cells. 48 h later, cells were subjected to selection with 50 μg/mL of hygromycin. The positive clones, which over expressed FLAG-tagged PCSK9 were detected by Western blot. FLAG-tagged protein IP was carried out by using FLAG-tagged protein immunoprecipitation kit (FlagIPT-1, Sigma, Dorset, UK) with final elution using 3xFLAG peptide (final concentration 150 ng/μL of 3xFLAG peptide). MYC tagged protein was immunoprecipitated with Pierce Mammalian c-Myc Tag IP/Co-IP kit (Waltham, MA, USA). PCSK9 proteins from the tissue culture mediums of the stable expressed 293 cell lines, including wild-type, D374Y and S127R PCSK9 were purified using FLAG-M Purification Kit (Sigma, Dorset, UK) following the manufacturer’s instruction. The final elution was concentrated with vivaspin 6 (Artoris Stedim Biotech, Surrey, UK) and dialysed against PBS using a Slid-A-lyzer mini dialysis unit (Thermo Scientific). Protein concentration was measured using Bio-Rad Protein Assay kit, Cat:500-0006, Bio-Rad. Hemel Hempstead, UK). The protein purity was determined by SDS-PAGE and visualized by Coomassie Blue stain with over 90% purity.

### 3.4. Shotgun Analysis of the FLAG-Tagged PCSK9 and Associated Proteins Complex Samples

A stable expressed FLAG-tagged PCSK9 cell line was grown in DMEM medium with 10% FBS. The FLAG-tagged PCSK9 protein was isolated using the FLAG-immunoprecipitation kit (Sigma, Dorset, UK) following by elution with the 3xFLAG peptide (final concentration 150 ng/μL of 3xFLAG peptide). The elution samples were then sent to the Protein Analysis Facility, Center for Integrative Genomics, Faculty of Biology and Medicine, University of Lausanne, Lausanne, Switzerland, where protein mixtures were separated by limited electrophoresis after which 3–5 molecular weight regions were cut and digested. Analysis was performed by LC-MS/MS on every fraction. The resulting spectra were pooled for every sample before a database search. The lists of identified proteins for each sample with their scores were subjected to statistical validation and aligned for comparison with Scaffold program. A negative control cell line, T-Rex-293 cells transfected with the empty vector pcDNA3.1 was used for background subtraction.

### 3.5. RNA Interference

c-IAP1 siRNA mix (pGB*-cIAP1*siRNA, Biovision Research Products, Milpitas, CA, USA) or control *siRNA* (*pGB*-control) were transfected into a stable wild-type PCSK9 overexpression cell line using the superfect reagent (Qiagene, Crawley, UK). After 48 h, the cells were subjected G148 selection to obtain the stable cell lines with nearly 100% knock-out the c-IAP1 protein as determined by Western blot analysis.

### 3.6. Mouse PCSK9 Immunoassay

The cell culture supernatants (from 1 × 10^7^ cells in T75 flasks) from c-IAP1 null mouse embryonic fibroblasts (MEFs) and matched wild-type MEFs cells [11] were collected. The mouse PCSK9 immunoassay kit (Cat MPC900, R&D system, Minneapolis, MN, USA) was used to quantitatively determine the PCSK9 concentration in cell culture supernatants according to the manufacturer’s recommended protocol. To ensure equal numbers of cells were used, the protein concentration of cell lysis was measured using the Bio-Rad protein assay kit (Bio-Rad, Hemel Hempstead, UK).

### 3.7. *In Vivo* Ubiquitination Analysis

pcDNA.3-Empty vector or HA-tagged ubiquitin plasmid (kindly provided by Edward Yeh [12]) and pRK5-HA-Ubiquitin-K27 (kindly provided by Sandra Weller [30]) were co-transfected with FlAG-PCSK9wt or FLAG-PCSK9-D374Y expression vector with or without the pcDNA6–c-IAP1 plasmids into T-REX 293 cells. 24 h later, the transfected cells were treated with MG132 (10 μM) for 1 h before the lysates were immunoprecipitated using the FLAG-immunoprecipitation kit (Sigma), the elution samples were then analyzed by Western blotting with anti- HA-Tag polyclonal antibody (Clontech, Mountain View, CA, USA).

### 3.8. *In Vitro* Ubiquitination Assay

Purified FLAG-tagged wild-type PCSK9 (4 μg) and D374Y-PCSK9 (1 μg) and recombinant c-IAP1 (1 μg, R&D System) were incubated in a reaction buffer (50 mM Tris-HCl (pH 7.5), 5 mM MgCl_2_, 2 mM ATP, 0.6 mM DTT) with recombinant rabbit E1 (100 ng), UbcH5b (250 ng) and ubiquitin (6 ng) at 37 °C for 2 h. The resulting mixtures were analyzed by immunoblotting using anti-PCSK9 antibody.

### 3.9. Mass Spectrometric Analysis of Ploy-Ubiquitinated PCSK9

Five μg of PCSK9-wild type protein was ubiquitinated *in vitro* as above. The poly-ubiquitinated PCSK9 bands were cut in small pieces and washed 2× with 100 μL of 100 mM NH_4_HCO_3_/50% acetonitrile, washed 1× with 50 μL acetonitrile. Gel slices then were digested with trypsin overnight. Samples were dissolved in 15 μL 0.1% TFA. 2 × 4 μL of each sample were added on the HPLC column and the spotted on a plate. The plates were analyzed in an automatic warp-run. The LC/MALDI/MS/MS measurements were performed automatically. Database searches were performed by using the Mascot (NCBI: all species) search program including check for possible contact lysine residue to the ubiquitin. This is the remaining molecule part of the ubiquitin part after trypsinisation (GG) (LRGG) [13].

## 4. Conclusions

c-IAP1 has two roles in regulating PCSK9 activities. The first role is that by binding to PCSK9, it promotes its maturation from the proprotein form to the mature form. The second role of c-IAP1 is its E3 ligase activity in ubiquitinating PCSK9. The finding indicates the c-IAP1 acts on both secretion of PCSK9 and its lysosomal localization.

## Supplementary Materials

Supplementary materials can be accessed at: http://www.mdpi.com/1420-3049/17/10/12086/s1.
