# Supplementary material for: c-IAP1 Binds and Processes PCSK9 Protein: Linking the c-IAP1 in a TNF-α Pathway to PCSK9-Mediated LDLR Degradation Pathway
Source: Molecules. 2012 Oct 15;17(10):12086–101. doi: 10.3390/molecules171012086 (PMC6268524; doi:10.3390/molecules171012086)

Support Information

Figure S1. SPR analysis of interactions of PCSK9 and c-IAPI. Representative overlays for various concentrations of purified PCSK9-wt (whole cell extract) to immobilized c-IAPI. Colored lines represent data; black lines indicate a theoretical good fit to a simple I: I kinetic model with a Kd of 44.3 ± 5 nM (n = 3).

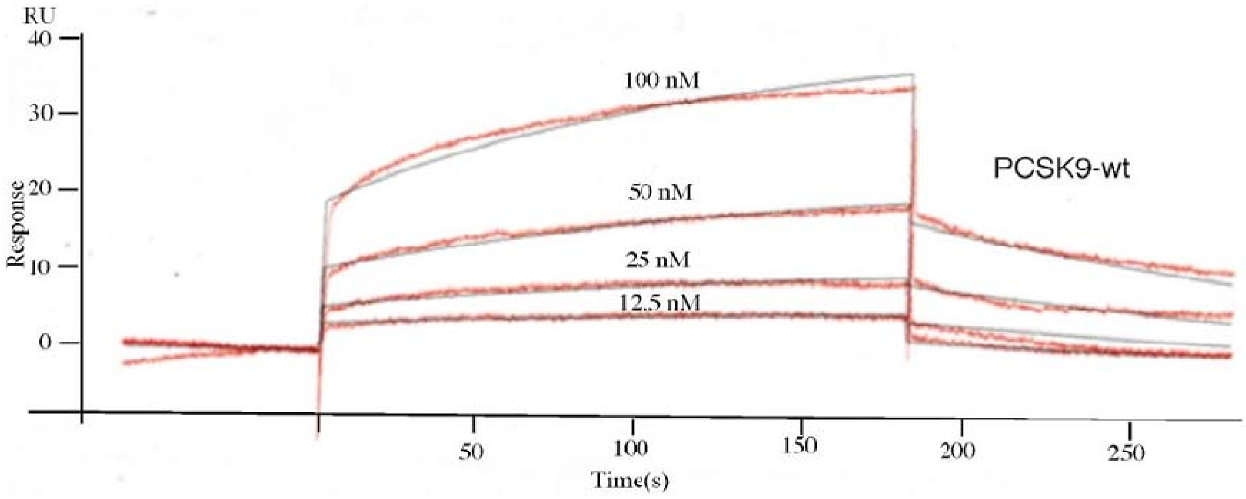

Figure S2. Co-localisation of PCSK9 and c-lAP1 in the cytoplasm. Cells stably overexpressing PCSK9 were immunostained with mouse anti-FLAG M2 monoclonal antibody (for detection of PCSK9) using secondary anti-mouse antibody labeled with Alexa488 (Green); Rabbit anti c-lAP was used to detect the c-lAP1. Protein, with an anti -rabbit-Cy3 labeled antibody. Cells were subjected to confocal microscopy examination. *Green* fluorescence indicates PCSK9; *red* indicates c-lAP1. In the merged images, *yellow* staining indicates co-localisation. Bar, 20-µm.

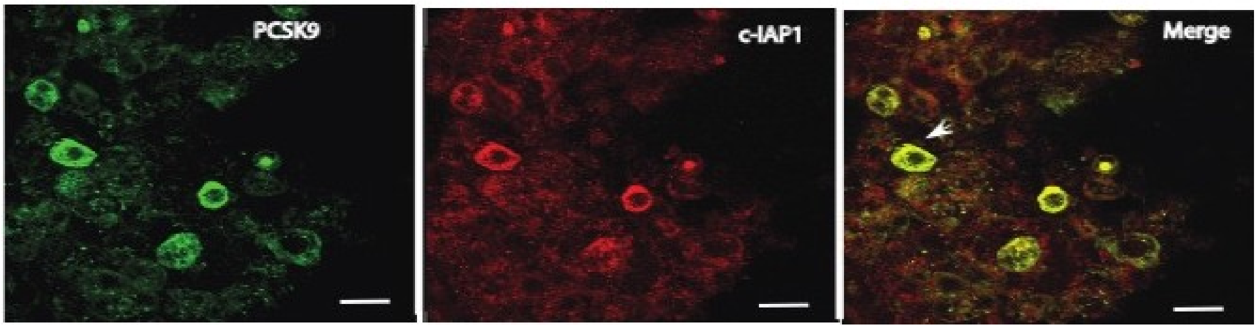

Figure S3. Mascot search result of LC/MALDI/MS/MS method with trypsin-digested poly-ubiquitinated wild- type PCSK9 bands, including check for possible contact lysine residue to the Ubiquitin, that is the remaining molecule part of the ubiquitin part after trypsinisation (GG) (LRGG).

Ubiquitin: Amino Acids
MW:8559.617
MQIFVKTLTGKTITLEVEPSDTIENVKAKIQDKEGIPPDQQRLIFAGKQLEDGRTLSDY
NIQKESTLHL
VLRLRGG
Digest Results
Search Parameter: MS Tol.: 50.000000 ppm, MSMS Tol.: 0.800000 Da, Trypsin Specific cleavages only
Sequence Editor
Tree hierarchy Meas. M/z Calc. MH+ Int. Dev.(Da) Dev.(ppm)
Range P Sequence peak 7 781.423 781.428 6889.480 -0.005 -6.143
1 - 6 0 MQIFVK 1: Oxidation (M) peak 16 1039.522 1039.517
13376.755 0.006 5.312 34 - 42 0 EGIPPDQQR
peak 37 1523.793 1523.781 97281.522 0.012 7.933 30 - 42 1 IQDKEGIPPDQQR
peak 60 1787.920 1787.927 2094.650 -0.008 -4.213 12 - 27 0 TITLEVEPSDTIENVK
peak 83 2402.199 2402.266 908.099 -0.067 -27.763 7 - 27 1 TLTGKTITLEVEPSDTIENVK
21: GG K27 Ubiquitination

Figure S4. PCSK9-meidated degradation of LDLR in mouse MEF cells. Immortalized MEFs derived from wild-type mice were culture for 16 h in non-serum DMEM medium treated with different amount of recombinant wild-type PCSK9. Immunoblot analysis of whole cell extract was carried out with anti-mature LDLR antibodies after 16 h treatment with purified PCSK9 protein. The results represent 3 independent experiments.

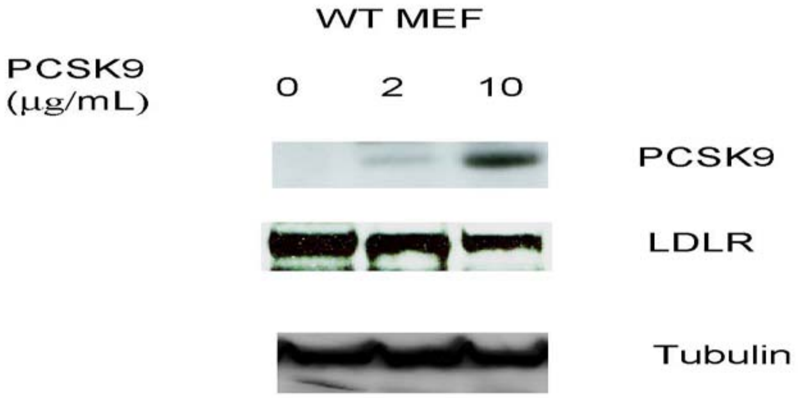

Figure S5. IP/western blot analysis of interaction between the FLAG-tagged PCSK9 and Stubl protein in PCSK9-FLAG pull-down assay. Cellular extracts from the T-Rex 293 stable cell line overexpressing FLAG-tagged wild-type PCSK9 and S 127R were subjected to anti-FLAG IP, blotted to nitrocellulose and probed with the Stubl antibody. 5% of the cell lysates prior to IP were used for the input, probed with anti-α-tubulin antibody. The western blots shown are representative of three separate experiments.

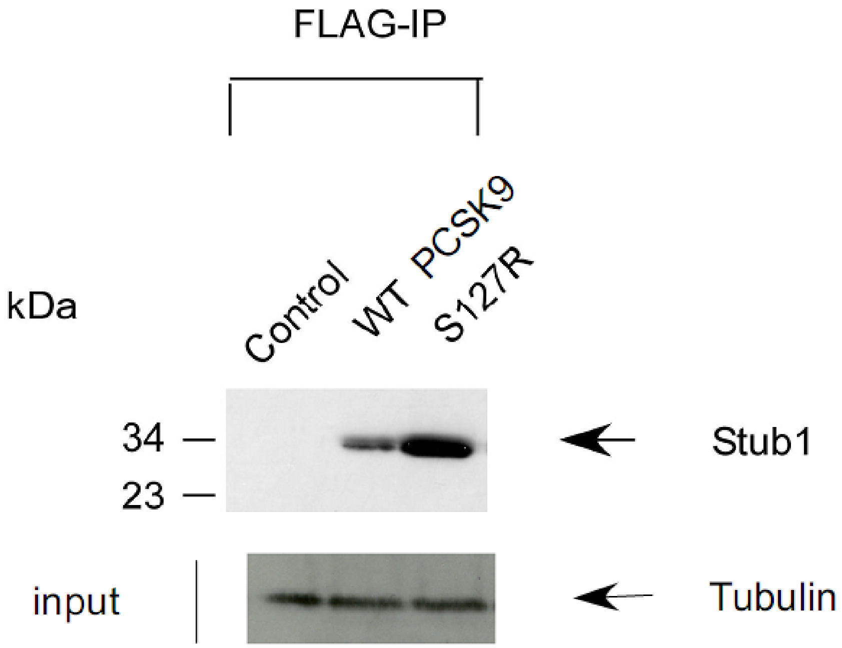

Supplement: Supplementary file 1 [file molecules-17-12086-s001.pdf]
